# What evidence supports the use of Body Worn Cameras in mental health inpatient wards? A systematic review and narrative synthesis of the effects of Body Worn Cameras in public sector services

**DOI:** 10.1111/inm.12954

**Published:** 2021-12-08

**Authors:** Keiran Wilson, Jessica Eaton, Una Foye, Madeleine Ellis, Ellen Thomas, Alan Simpson

**Affiliations:** ^1^ Health Services and Population Research Department Institute of Psychiatry, Psychology & Neuroscience King’s College London London UK; ^2^ Great Ormond Street Hospital for Children London UK

**Keywords:** body worn cameras, de‐escalation, mental health, public sector services, security

## Abstract

Body‐Worn‐Cameras (BWCs) are being introduced into Mental Health Inpatient Units. At present, minimal evidence surrounding their use in a mental health environment exists. This review examined research on the uses of BWCs in public sector services including healthcare, public transportation, and law enforcement. All eligible studies included a visible BWC, recording on a continuous loop as the main intervention. The evidence base presented high levels of bias, highly varied camera protocols, and heterogeneity of outcome measurements. This review found there is limited evidence for the efficacy of BWCs to control and manage violence within mental health inpatient wards. The technology has shown to be effective in reducing the number of public complaints in a law enforcement setting, but it is unclear how this is achieved. It appears there may be potential beneficial uses and unintended consequences of BWCs yet to be explored by mental health services.

## INTRODUCTION

The use of digital technology in healthcare settings has increased over recent years and presents new opportunities for the delivery of physical and mental health services in the United Kingdom (Department of Health and Social Care 2019). In 2005, CCTV was first introduced as a technological surveillance tool for maintaining staff and patient safety in a range of healthcare settings (Desai 2009). Continued technological advances in this field have led to the development of wearable camera technologies referred to here as Body Worn Cameras (BWCs). These small transportable devices are usually worn on the outside of clothing, glasses, or headwear to produce video and audio recordings (The Metropolitan Police, 2020).

This technology has been in use by police in the UK since 2005 (The Home Office 2007) and it is estimated that over 70% of police forces have started to adopt the use of BWCs (Lum *et al*. 2020). In a police setting, research suggests BWCs may enhance transparency, supplement documentation, and deter illegal and inappropriate behaviours from both officers and citizens (Bureau of Justice Assistance 2015). However, there is a lack of evidence supporting their efficacy, cost effectiveness, and wider social impact. The only comprehensive systematic review of literature to date found that the use of BWCs by police officers had no significant impact on police (mis)use of force or assaults against officers (Lum *et al*. 2020).

Despite a lack of evidence to support the use of BWCs in public sector services, The National Health Service (NHS) has pledged to invest (UK) £8 million in pilot testing BWCs as a way to enhance staff safety and assist in prosecuting violence against staff (Department of Health and Social Care 2019). Since this announcement, we have seen the roll out of BWCs in ambulance services across the country (London Ambulance Service 2021) and initial pilot testing in inpatient mental health services (Ellis *et al*. 2019). Safety is a considerable issue within mental health services, where staff are 7.5 times more likely to report they have been attacked than staff in other NHS services (Royal College of Nursing 2018b). In 2020, 14.9% of staff in mental health trusts claimed that they experienced physical violence from service users or other members of the public (NHS Staff Survey 2020: National Results Briefing 2021). Despite the higher rates of physical assault against staff, only 4% of alleged physical assaults by psychiatric inpatients were reported to the police in 2018 and even fewer resulted in action taken (Doedens *et al*. 2020; Young & Ready 2016). In a mental health setting, BWCs allow staff to record situations where conflict may occur, and containment measures may be used. However, the use of BWCs in mental health settings is in its infancy and is a strongly contested intervention (Royal College of Nursing 2018a).

The high rate of assault against mental health nurses contributes to the high rate of staff turnover in mental health services (NHS Improvement 2019; Royal College of Nursing 2018b). Despite identifying the staffing crisis as a priority in the Five Year Forward View 2017–2019 report (Department of Health and Social Care 2017) and the NHS Long Term Plan (Department of Health and Social Care 2019), the number of mental health nurses increased by <0.5% over 2019 (Buchan *et al*. 2019) and recruitment and retention remain huge challenges (Launder 2020). Understaffing plays a key role in the deterioration of patient care (Baker *et al*. 2019), and BWCs may be implemented in an attempt to improve staff safety and ultimately improve patient care.

However, conflict and violence in mental health wards is a complex issue that affects both staff and patients (Bowers 2014; Fletcher *et al*. 2021; Kumar *et al*. 2001). Research and audits conducted in inpatient services in England revealed patients often experience verbal abuse, fighting, bullying, theft, racism, and sexual assault (Care Quality Commission 2018; Jones *et al*. 2010). Mental health staff have a responsibility to protect their patients from physical and psychological harm during their stay; yet, frequently staff interactions with patients can fuel conflict (Papadopoulos *et al*. 2012). Mental health nurses are the staff group most likely to be involved in face‐to‐face interactions with service users who may be highly distressed and/or frustrated by the restrictions typically imposed within inpatient settings to maintain safety, such as removing personal items, restricting smoking, or limiting movements (Bowers *et al*. 2015). There are currently evidence‐based interventions, such as the Safewards model, which have been found to reduce incidents of conflict and use of containment measures (e.g., seclusion, restraint) on mental health wards (Bowers 2014). However, chronic understaffing can make it difficult to implement these effective interventions more widely (McAllister *et al*. 2019).

Ethical concerns remain central to wider debates surrounding the implementation of BWCs in mental healthcare settings (Royal College of Nursing 2018a). Those who require care in an inpatient mental health ward are often admitted against their will and are at the most vulnerable point in their patient journey (Care Quality Commission 2019). Further, Black and South Asian minority groups are disproportionately detained under mental health legislation in the UK, and Black patients are more likely to be subject to measures to maintain safety, such as use of physical restraint (Barnett *et al*. 2021; Payne‐Gill *et al*. 2021; Rodrigues *et al*. 2020). Implementation of BWCs would likely have the greatest impact on this already vulnerable group.

While policy makers hope this new technology will bring improvements to the delivery of mental health services, it is essential that patients receive care based upon the best current evidence in conjunction with clinical expertise and patient values (Reid *et al*. 2017). A small number of BWC evaluations in mental health wards in England have been undertaken (Ellis *et al*. 2019; Hardy *et al*. 2017), but given their relatively small scale and localised focus, a wider review is required. The lack of research on BWCs in a mental health setting means it is essential to draw upon the wider literature in the public sector to explore its effects and consequences. A systematic review provides the opportunity to appraise and synthesise existing evidence across the public sector and make recommendations regarding the potential future use of BWCs in a mental health environment (Pati & Lorusso 2018).

## AIMS

This review seeks to answer the research question: Are BWCs likely to enhance safety in mental health inpatient wards based on the literature regarding BWC use across a range of public sector services*?* The primary objective is to identify and systematically review literature relating to the use of BWCs in the public sector, assessing where/how BWCs are being deployed, the methods used to conduct such research, and the effect of BWCs in these settings. The findings and implications associated with BWCs in public sector services will be discussed in relation to their suitability for mental health services.

## METHODS

### Design

We conducted a systematic review using narrative synthesis in accordance with the Guidance on the Conduct of Narrative Synthesis (Popay *et al*. 2006). This approach facilitates synthesis of a range of methodologies and study designs and allows a focus on the wide range of BWC applications in public services. A meta‐analysis of this literature was not a suitable way to address the research questions proposed in this review (Borenstein 2009). For the purposes of this review, public sector services are defined as central government, local government, and public corporations delivering services to citizens, including healthcare, law enforcement, and public transportation (Office for National Statistics 2019).

### Protocol and registration

The review protocol was registered with PROSPERO before commencement (CRD42020164878).

### Eligibility criteria

Studies were only included where they met the following criteria: BWC was a standalone, visible device able to provide continuous video playback; BWC was attached to a member of staff working in a public sector service; BWC was used to record face‐to‐face interactions with the public or service users; and the study reported the methodology for evaluation. Studies were excluded if the BWC was used as a data collection tool; BWC was used outside the public sector; or BWC use was reported in an internal evaluation without reporting the methodology.

### Database coverage

A search of all relevant literature was undertaken with librarian assistance using the following databases: Medline (via Ovid, 1966 to 25/05/2021); Embase (via Dialog 1974 to 25/05/2021); PsycINFO (via Datastar 1806 to 25/05/2021); Global health (via Ovid, 1966 to 25/05/2021); HMIC (via Ovid, 1979 to 25/05/2021); Web of Science (via Clarivate 1975‐ 25/05/2021); Sage Journals (via Datastar 1994‐ 25/05/2021); OpenGrey (1972 to 25/05/2021) and Google Scholar (2004‐ 25/05/2021). No limits or filters were imposed. Searches were supplemented by reference list screening and BWC websites were reviewed for publications (Reveal, Axon, Google Glass, Calla, VIEVU, Panasonic and Puma). Email alerts from all journals and RSS feeds from camera websites were utilised to ensure identification of new articles.

### Search strategy

A keyword search was applied to all databases using the search terms; Body adj3 camera* OR Wearable video OR Wearable camera* OR Body worn video. An initial scoping search revealed most journal articles had yet to be indexed under a MeSH term, therefore, MeSH terms were omitted from the facet analysis.

### Study selection

**FIG. 1:**
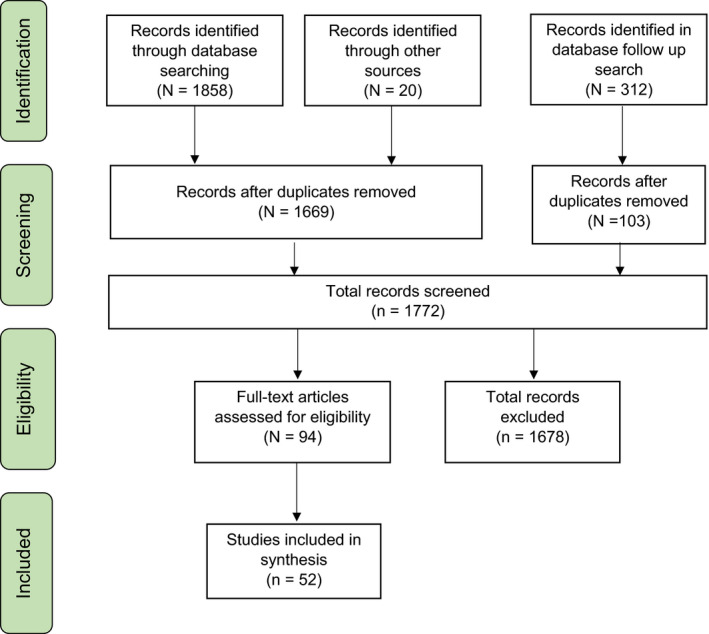
PRISMA flow diagram (Moher *et al*. 2009).

In May 2019, the search was conducted by JE. In May 2021, the search was repeated by KW to capture new research that was published in the time elapsed since the initial search. PRISMA reporting guidelines were followed, and a full PRISMA chart can be found in Figure 1. Referencing software Zotero was used to de‐duplicate, and articles were screened in Microsoft Excel. To ensure unbiased selection, title and abstract screening was undertaken by two independent researchers (First search: JE, UF; Second search: KW, UF). Full text screening utilising the same criteria was undertaken. Full text exclusions were documented along with reasons. Disagreements at any stage were resolved by a third reviewer (AS; *n* = 6).

### Data collection

Data was extracted using a pre‐designed data collection tool included in the PROSPERO registration. The tool includes country, study design, sample size, setting, main method, camera user, recording subject, camera model, comparison arm, study length, outcome measures, and funding source. The form was piloted on 10 studies during the initial search to ensure suitability. No amendments were made. No limit on summary measures were implemented and all relevant results were collected. Efforts were made to contact authors for missing information wherever possible.

### Quality appraisal

All studies were reviewed for selection, performance, attrition, detection, and publication bias. The Joanna Briggs Institute (JBI) Critical Appraisal tool was utilised (Aromataris & Munn 2020). Critical appraisal of all included studies was undertaken individually by JE and UF. No discrepancies were apparent. Individual scores attained from the tool were then collated to review bias across studies.

### Analysis

Results were divided into healthcare, law enforcement, and public transport sectors and analysed using a narrative synthesis approach according to an established framework (Popay *et al*. 2006). With this approach, main findings from the quantitative studies in each sector were summarised and further supplemented with examples from the qualitative studies. KW, JE, and ME identified the effects, outcomes, and experiences of BWCs from the qualitative data.

## RESULTS

### Study characteristics

An overview of the 52 studies included in this review can be found in Appendix A. Most were conducted in law enforcement settings (including police officers, prison guards, and traffic wardens; *N* = 43), followed by healthcare (including physicians, frontline clinical staff, paramedics, and family carers; *N* = 8) and transportation (railway ticket inspectors; *N* = 1). There was a wide range of study designs, such as randomised control trials, pilot evaluations, and qualitative interviews; however, not all studies explicitly reported their chosen design.

#### Participants

BWC research is comprised of various units of analysis reported alongside participant population data. A total of 10 articles reported the number of cameras used in the study (7 in law enforcement, 2 in healthcare, 1 in public transportation). Participant figures are more commonly reported than number of BWCs due to frequent sharing of a limited number of cameras across entire departments. Across all studies, 46 reported a specific count of participants (6 in healthcare, 39 in law enforcement, 1 in public transportation). Other units of measurement reported in lieu of participants or number of cameras include shift patterns (Ariel *et al*. 2015), contacts or interactions with police (Young & Ready, 2016), geographic regions (Grossmith et al., 2015; Mitchell *et al.* 2018), caregiving pairs (Matthews *et al*. 2015), and physician consultations (Gupta *et al*. 2016).

#### BWC intervention

The model of camera used also varied widely across the studies. Police studies primarily used TASER cameras; however, 23 of these studies neglected to declare a model. One study (Ho *et al*. 2017) used TASER cameras in a healthcare setting with paramedics. However, Google Glass (4) and Calla (2) were most common in healthcare settings. There was also variability in protocol reporting. Only 17 out of 52 (32%) studies reported recording protocols, and these were primarily law enforcement. The practice of camera wearers verbally announcing recording varied and a range of visual warnings to alert others varied from audible sounds, flashing lights and activation of screens. Camera activation was either at staff discretion or mandatory for the duration of a shift.

## Research quality

All studies were reviewed for selection, performance, attrition, detection, and publication bias. The JBI Critical Appraisal tool was utilised. Critical appraisal of the included studies was undertaken by three researchers (JE, UF, KW). No discrepancies were apparent. Individual scores attained from the tool were then collated to review bias across studies and assess quality. Overall, 30 out of the 52 studies included in this review were of poor quality, with a high risk of bias. Only five studies displayed low enough risk to be classified as good quality research. Quality assessment can be found in Appendix A.

### Funding and costs

A total of 19 studies reported funding sources. A further six studies did not receive funding, and the remaining 30 studies neglected to report on funding sources. Of the 19 reports of funding, 8 were funded by a federal government body (e.g., Bureau of Justice, Home Office, US Department of Justice), and 4 were funded by other government bodies such as local task force and police budgets. Five studies (Ariel *et al*. 2015; Ariel 2016b; Ellis *et al*. 2019; Hardy *et al*. 2017; Mitchell *et al*. 2018) reported receiving camera equipment free of charge from the company. Overall, law enforcement studies account for 80% of studies with undeclared funding.

Three studies (Braga *et al*. 2018; Hardy *et al*. 2017; ODS Consulting 2011) report on the total costs of BWC implementation, including hardware, software, and training. On average, the cost per BWC was £1,750. However, none of the studies in this review included an economic analysis in which wider consideration of resource use, training costs, health benefits, and opportunity costs can be calculated and analysed.

### Outcomes in transportation sector

Only one study examined the use of BWCs by staff at railway stations across England (Ariel *et al*. 2019). The results from this randomised controlled trial indicated a 47% reduction in odds of assaults against staff when wearing BWCs. This article positions BWCs as beneficial to employee health and safety by reducing rates of violence against staff.

### Outcomes in law enforcement sector

Most studies included in this review were from law enforcement settings (43 out of 52). Twenty of those studies examined the impact of BWCs on one or more of the following outcomes (Table 1): police behaviour (use of force, arrest rates), citizen behaviour (complaints, assault against officers). Combinations of these four variables were the most frequently examined outcomes. Additionally, 12 studies examined both civilian and police opinions on BWCs. Other less frequently examined outcomes were court processes (Morrow *et al*. 2016; Owens *et al*. 2014), traffic stops (Peterson *et al*. 2018), stop and frisks (Young & Ready 2016), response time/time spent on scene (Wallace *et al*. 2018), camera activation (Roy 2014; Young & Ready 2016), and public reporting of crime (Ariel 2016a).

**TABLE 1:** Impact of BWC on police use of force, arrest rates, citizen complaints, and officer assaults

Paper	Use of force	Arrests	Complaints	Assaults
Ariel *et al*. (2015)	−61.4%***	–	−91.3%**	–
Ariel (2016b)	No significant change	–	–14%*	–
Ariel *et al*. (2017)	–	–	−93%***	–
Ariel *et al*. (2018)		–	–	−61%*
Braga *et al*. (2018)	−40.7%*	+ 5.2%**	−30.2%*	–
Braga *et al*. (2019)	–63.6%*	–	–50.5%*	–
Ellis *et al*. (2015)	–	–	−15%†	–
Gaub *et al*. (2020)	No significant change	–	–	–
Groff (2020)	−38.3%***	–	−39.2%***	–
Grossmith *et al*. (2015)	–	No significant change	No significant change	–
Headley *et al*. (2017)	–	−16.1%*	–	No significant change
Hedberg *et al*. (2017)	–	No significant change	−96%***	–
Huff *et al*. (2020)	+0.10%**	+ 34.8%**	No significant change	–
Jennings *et al*. (2017)	−8.4%†	–	−65.4%†	–
Rankin (2013)	–75%†	–	–40%†	–
Mitchell *et al*. (2018)	–	–	−86%***	–
Morrow *et al*. (2016)	–	+ 6.6%*	–	–
ODS Consulting (2011)	–	–	–	Decrease†
Ready and Young (2015)	–	−6.9%*	–	–
Peterson *et al*. (2018)	No significant change	No significant change	No significant change	No significant change
Pope *et al*. (2020)	No significant change	No significant change	–	–
Wallace *et al*. (2018)	–	No significant change	–	–
White *et al*. (2018)	No significant change	–	No significant change	–
Yokum *et al*. (2017)	No significant change	–	No significant change	–

*p < 0.05; **p < 0.01; ***p < 0.001.

– Not measured.

^†^ Significance level not reported.

#### Officer Behaviour

Two studies reported an increase in officer‐initiated contact (Wallace *et al*. 2018; Young & Ready 2016), and a third reported a decrease (Huff *et al*. 2020). Although (Young & Ready 2016) reported an increase in officer‐initiated contact, they also reported that officers were less likely to perform stop and frisks while wearing BWCs. However, BWCs were not found to impact response time or time spent on scene once contact was initiated (Wallace *et al*. 2018). Research also shows a mix of statistically significant increases (Braga *et al*. 2018; Huff *et al*. 2020, Morrow *et al*. 2016) and decreases (Headley *et al*. 2017; Young & Ready 2016) in arrest rates, and one study reported no change at all (Wallace *et al*. 2018). No conclusions can be drawn about the impact of BWCs on officer‐initiated contact or arrest rates based on this literature.

Four studies reported significant decreases in police use of force resulting from BWC use (Ariel *et al*. 2015; Braga *et al*. 2018, 2020; Groff 2020). Two further studies also reported a slight decrease in use of force but neglected to report the statistical significance of this change (Jennings *et al*. 2017; Rankin 2013). Additionally, one study reported a decrease in use of force rates only when controlling for compliant handcuffing (Henstock & Ariel 2017). However, (Huff *et al.* 2020) reported a small but significant increase in use of force following BWC activation. There appears to be a trend toward decreased use of force after implementing BWCs, but inconsistencies in methods and measures across the literature make it difficult to draw solid conclusions.

#### Officer opinions

When asked about BWCs, the ten studies examining police perspectives report mixed opinions. Some studies have found police officers believe the cameras are helpful and should be implemented (George & Meadows 2016; Pelfrey & Keener 2016; Ready & Young 2015), while others report neutral and negative leaning opinions (Hyatt *et al*. 2017). For example, officers in one study believed that BWCs enhance the quality of evidence, particularly in prosecuting domestic violence cases (Gaub *et al*. 2016). However, another study reported contradictory findings in which officer perspectives on BWC helpfulness for interpersonal violence prosecution was less favourable after BWC implementation (Morrow *et al*. 2016).

Qualitative studies examining police officer perspectives highlighted some of the perceived benefits of BWCs. One common belief was that BWCs can protect officers against citizen complaints:

You give the command, the dog pops off and comes back to you, and that’s captured on camera. I mean that’s gold to us. Later down the road if there is any civil litigation, it’s there. It’s captured for the argument that the dog stayed on too long. Canine Officer (Gaub *et al.* 2020)

…don’t think they will create any problems for the officers although they may hold some officers to a higher standard of professionalism, will assist in stopping complaints about officers if they occur. Police officer (Makin 2016)

Officer beliefs about the impact of BWCs on civilian behaviour were mixed. One study found that while some officers believed the cameras would benefit the officers, they did not believe the cameras would enhance officer safety or change civilian behaviour (Pelfrey & Keener 2016). This contradicts the results of another police survey in which officers reported beliefs that BWCs do change civilian behaviour (George & Meadows 2016). Similarly, a BWC pilot evaluation in the UK found that prison staff showed increased perceptions of safety after BWC implementation (Pope *et al*. 2020).

There have also been contradictory findings on whether police officers feel the implementation of BWCs has impacted their own behaviour. One qualitative study demonstrates possible positive behaviour changes resulting from BWC use:

This was a pretty straightforward situation. But when you know, you are having a bad day, or you are in an intense situation you know? I would normally maybe let a curse word fly. And we are not allowed to curse at citizens, but you know, we all do that from time to time. Now, I tend to watch myself a little more. Patrol officer (Koen et al. 2019)

However, George and Meadows (2016) found that officers believed the cameras would improve the behaviour of their colleagues, but not their own.

These studies also highlighted perceived challenges and limitations to BWC use. Volume of calls, technical limitations of recording and battery capacity, and documentation procedures were all identified as problems (Gaub *et al*. 2020). Another concern raised by officers was that BWCs would be used as a tool to control and discipline the officers:

Regardless of statements used by management, the primary use of these videos will be to police the officers’ actions and to be used for disciplinary actions.Police officer (Makin 2016)

In addition to concerns about BWCs being used to discipline officers, another officer raised a concern about officers manipulating footage:

Cameras have limited abilities and perceptions and could be used negatively by outside influences to create problems. How many times have you seen video where the clips were cut to create the perception someone wanted to portray, rather than the entire circumstance? Police officer (Makin 2016)

Ultimately, officer opinions appear to be the most robust and widely researched aspect of BWC implementation in law enforcement. The opinions of officers vary widely, and qualitative research has captured this in detail.

#### Citizen behaviour

Rates of citizen complaints were frequently reported alongside police use of force and arrest rates. Despite being a measure of citizen activity, complaints are most frequently operationalised as a measure of officer behaviour. Ten studies reported decreases in citizen complaints; however, three of those studies neglected to report statistical significance of that decrease (Ellis *et al*. 2015; Jennings *et al*. 2017; Rankin 2013).

Methods for reporting changes in officer assaults varied across the five studies which examined this outcome. A pilot study of BWC use by a police force in Scotland reported a decrease in assaults with no statistics to support this statement (ODS Consulting 2011). Another study also reported a significant difference (Ariel *et al*. 2018), but this is contradicted by an earlier study which reported an increase in assaults against officers (Ariel *et al*. 2016). A further two studies reported no significant changes (Headley *et al*. 2017; Peterson *et al*. 2018). Additionally, one study reported assaults by comparing the frequency of change in assault rates per prison, rather than reporting specific changes in actual assault rates (Pope *et al*. 2020); therefore, this study cannot be directly compared to studies that reported figures for assaults. This study reports that prison sites with BWCs experienced fewer officer assaults after implementation; however, this was statistically insignificant. It is unclear from the existing literature whether BWCs have an impact on assaults against officers.

#### Citizen opinions

When asked how BWCs would impact police behaviour and outcomes, 99.4% of citizens surveyed in one study supported police use of BWCs. Citizens surveyed in this study also believed BWCs would improve the quality of police behaviour, increase lawfulness and transparency, and reduce corruption (Demir 2019). When BWCs were present in another quasi‐experimental study, citizens perceived the officer’s behaviour more positively, and ultimately perceived police as more legitimate (Demir *et al*. 2020). However, prisoner interviews conducted during a pilot evaluation of BWCs in UK prisons reported less favourable opinions about BWCs, and a perception that staff/prisoner relationships were negatively affected following implementation (Pope *et al*. 2020). Ultimately, prisoners felt less safe after BWC implementation and perceive the cameras as a violation of privacy:

It is another intrusion of the little privacy I had left. Prisoner (Pope *et al*. 2020)

Like the concerns raised by police officers about officer manipulation of footage, prisoners also raised concerns about how staff are using the BWCs:

Body worn cameras are not being used as it should be. It is being used just to set up prisoners for nicking. Prisoner (Pope *et al*. 2020)

While officer perspectives on BWCs have been researched in some depth, research on citizen and prisoner perspectives is far less robust.

### Outcomes in healthcare sector

Five studies examined BWCs in emergency settings, including surgical consultations (Gupta *et al*. 2016), poisoning assessments (Skolnik *et al*. 2016), stroke assessment (Noorian *et al*. 2019), paramedic activity (Ho *et al*. 2017), and trauma triage (Broach *et al*. 2018). Findings indicate that diagnoses and assessments made with BWC technology are just as reliable as those made in‐person (Broach *et al*. 2018; Noorian *et al*. 2019; Skolnik *et al.* 2016). However, these studies are quasi‐experimental and observational in design, with small sample sizes (≤10). Therefore, individual preferences, practice patterns or policy decisions can impact selection criteria for participants and outcomes cannot be attributed to the intervention alone (Carlson & Morrison 2009).

Further, one study used medical simulation to evaluate the use of BWCs for documenting paramedic call outs. The purpose of using BWCs in this study was not to improve quality of care or safety; rather, it was specifically to improve staff documentation of paramedic activity. At present, current Emergency Medical Services documentation practices are usually taken from memory after the event. A simulation of an unconscious patient was played out by role players to investigate whether the accuracy of documentation could be improved by retrospectively watching BWC footage of the scene. Watching BWC footage after callouts resulted in 71 changes to documentation regarding missing or incorrect information from paramedic reports; the authors only report raw data and cannot comment on the relationship between the variables. This research presented BWCs as a solution to improve and streamline current ways of working. The technology shows potential for improving documentation, but this research base is still developing.

In addition to emergency assessment and documentation accuracy, qualitative findings highlighted the importance of streaming footage for dementia patients who face barriers in accessing healthcare:

I just think that it could help the doctors, because…they don’t want to come into the house… And that, honestly, was the biggest problem – she went for a year without seeing a doctor in the moderate to late‐stage Alzheimer’s range…But this could help in that way…if it was forwarded to the doctor’. Family caregiver (Matthews *et al*. 2015)

#### Mental health

Only two studies included in this review examined the use of BWCs in mental health settings (Ellis *et al*. 2019; Hardy *et al*. 2017). Both studies reported on the use of BWCs in mental health settings, with one reporting from the north of England and one from the greater London area. The first (Hardy *et al*. 2017) was a feasibility study, which employed 12 cameras, provided free of charge by Calla, across five wards (two recovery, one low secure, one acute, and one intensive). This study reported an increase of verbal abuse and violence on three wards. A further two wards reported decreases in violence and low‐level restraint. However, two wards also reported increases in low‐level restraint. Finally, three wards reported a reduction in emergency restraint. It is important to note that these figures are only descriptive and have not been reported in relation to the type of ward. Therefore, it is not possible to make any claims about the impact of BWCs depending on level of ward security or admission type. [Correction added on 23 December 2021, after first online publication: the second sentence in the preceding paragraph has been amended and was originally “Both studies reported on the use of BWCs at the same NHS trust in the north of England.”]

Researchers also collected qualitative responses from staff and patients about their perspectives on the pros and cons of BWCs. Staff who wore cameras expressed positive perceptions:

‘I think it prevents lots of aggression and puts patients’ minds at ease knowing there is a record of what happened’. Staff member (Hardy *et al*. 2017)

However, staff who did not wear cameras expressed more mixed opinions:

‘They cause more problems because the responding staff will only capture from the time of arrival hence does not give a clear picture of what has been happening prior to that’. Staff member (Hardy *et al*. 2017)

Of the 57 patients surveyed, 68% felt the cameras would change staff behaviour and 63% felt it would change patient behaviour:

It may make staff more confident to approach and help distressed patients, it makes them feel safer at work so happier and more able to help patients. Patient (Hardy *et al*. 2017)

However, some patients expressed less favourable perspectives:

It causes patients to be more irritable and angry when they think they are being observed. Patient (Hardy *et al*. 2017)

The second study (Ellis *et al*. 2019) was a quasi‐experimental trial conducted two years after the feasibility study. This trial involved 50 cameras, again provided by Calla, across seven wards (two acute, one intensive, one forensic low secure, one medium secure, and two enhanced medium wards). This study reported on the context of each ward in more depth than the feasibility study, and also reported findings specific to the type of ward. The authors evaluated recorded incident data pre and post implementation of BWCs. Incidents were ranked by seriousness, ranging from 1 (verbal aggression) to 4 (restraint requiring tranquilising injection). Overall, a non‐significant decrease in incidents was reported, but a significant change in the seriousness of incidents across specific ward types was found. The two acute wards showed a significant increase in low level violence without restraint, and a decrease in incidents involving restraint with tranquilising injections. No difference was reported on the medium, intensive, or forensic wards. [Correction added on 23 December 2021, after first online publication: “at the same site” has been removed from “The second study.…feasibility study.” in the preceding paragraph]

Despite the low‐quality research and mixed findings, both studies report that BWCs are acceptable, beneficial, and effective tools in a mental health setting. It is important to note that both studies received cameras free of cost from the manufacturers, thus may be subject to bias. Ellis *et al*. (2019) provided a disclosure statement explaining that the lead author’s expenses were reimbursed by the camera company but maintained that the evaluation was conducted independently. Hardy *et al*. (2017) did not include a disclosure statement but did thank the camera company for providing cameras and training free of charge in the acknowledgements section of the article.

## DISCUSSION

The primary objective of this review was to evaluate public sector use of BWCs in order to inform judgement about their suitability in mental health services. Results from this review highlight that BWCs are being implemented and utilised for different purposes across the public sector. In medical and emergency healthcare settings, BWCs tend to serve an explicitly therapeutic purpose by aiding in virtual assessments, diagnosis, and documentation. Similarly, in dementia care wearable cameras are being utilised for remote care and assessments to aid the safety of patients. The healthcare literature included in this review indicates that BWCs are successfully being used for telehealth purposes in a variety of contexts. Despite the apparent success of BWCs in medical healthcare settings and the growth of tele‐mental healthcare before and during the COVID‐19 pandemic (Barnett *et al*. 2021; Mishkind *et al*. 2021), there appears to be less research focussed on the potential therapeutic value of BWCs in mental health settings and much more on its role in law enforcement and prevention of violence.

This review found that mental health services are beginning to use BWCs similarly to the law enforcement sector’s use of this technology to document and deter aggressive incidents. The similarities between BWC use in mental health and police settings indicates that mental healthcare aligns more with the narrative of control and coercion prevalent in policing, rather than the arguably more patient‐centred approach found in physical healthcare. However, this distinction is not clear cut; reducing aggression and assaults in mental healthcare settings helps create a safer, calmer therapeutic environment that benefits patients, aids the wellbeing and retention of staff, and reassures families. The question remains as to whether BWCs are an effective and acceptable method of achieving that while maintaining parity of esteem between mental and physical healthcare (Panday 2016).

Research into the use of this technology in mental health settings remains in its infancy as evidenced by the limited number and quality of papers found within this review (Ellis *et al*. 2019; Hardy *et al*. 2017). These early pilot studies of BWCs in mental health settings present significant limitations due to low quality design, urging caution in drawing any conclusions around the impact on staff and service user behaviour.

While there does appear to be a trend toward decreased police use of force after implementing BWCs, this review found that there are inconsistencies in reporting methods and operational definitions making it difficult to draw solid conclusions based on this literature, and thus calling into question the applicability of such evidence to mental health settings. The review also indicates a reduction in complaints against police officers; however, the evidence fails to address whether BWCs result in fewer false accusations of police misconduct, or whether it deters officers from exercising illegitimate use of force. It is also unclear whether BWCs have an impact on citizen assaults against officers. Therefore, this review indicates there is no generalisable research supporting the use of BWC to reduce patient violence against staff, which is the main motivation for implementation in mental health settings (Hancock 2018). Despite the large evidence base examining BWC use in law enforcement settings, it is unclear if or how BWCs may enhance safety for either citizens or police officers. The heterogeneity of the samples, study settings, and cofounding factors also means to draw conclusions on the use of BWCs in a mental health setting based on law enforcement outcomes would be naïve.

It is important to recognise the different environments in which police officers and mental health staff work. Most of the research examining BWCs in law enforcement settings rely on patrol officers interacting with members of the public on the street. Mental health wards are enclosed spaces in which the wearer often has an ongoing relationship with the person they are recording. Mental health staff have a duty of care to vulnerable patients and reliance on building and sustaining therapeutic relationships is arguably not as relevant in public police–citizen interactions. Such differences may not only have a confounding influence on the nature and outcome of BWC use in mental health settings, but they may also lead to unintended consequences.

Based on the very limited evidence from mental health settings, BWCs may decrease high‐level incidents and increase low‐level incidents of aggression on inpatient wards. However, using BWCs to change patient behaviour raises questions around technological coercion (Morris 2021). While there is very little evidence to date on the impact of technological coercion on patient behaviour, coercive tactics that place environmental controls around patient behaviour have been linked to adverse outcomes. Research indicates that self‐harm rates on inpatient wards rise when patients feel the nurses are controlling them or limiting their freedom (James *et al*. 2012). Interviews with prisoners indicate that BWCs create feelings of powerlessness and intrusion (Pope *et al*. 2020), and similar sentiments are echoed by inpatient service users (Hardy *et al*. 2017).

If BWCs do reduce patient violence in inpatient mental health settings, they could potentially allow mental health staff to engage in more directed therapeutic work with the knowledge that they are less likely to require strong coercive techniques, such as seclusion or restraint (Stewart *et al*. 2010). However, even well intentioned safety measures such as door‐locking can create feelings of imprisonment and resentment which impair attempts at creating a therapeutic environment (Muir‐Cochrane *et al*. 2012). It is important for researchers, policy makers, healthcare professionals, and indeed patients themselves, to ask whether technological coercion is better for patients than the evidence‐based measures already available, such as the well‐established Safewards model (Bowers 2014).

Discussions around the risk of BWCs exacerbating symptoms and compromising care in mental health settings have begun to emerge (Olive 2019; Royal College of Nursing 2018a), but there is a considerable lack of research into patient perspectives on BWCs. While officer perspectives on BWCs have been researched in some depth, research on citizen and prisoner perspectives is far less robust and consequently policy makers must acknowledge the bias in the current evidence base when considering the implications for mental health services.

### Limitations & future directions

This review is the largest, and only, study of its type to date. The synthesis of evidence across the public sector has provided a wide overview of the uses and effects of BWCs and examines the minimal evidence for the use of this technology in a mental health setting. This review has identified poor methodological rigour in the current BWC evidence base and a lack of generalisability to mental health settings. Future research must explicitly examine the impact of BWCs in mental health settings, taking both patient and staff perspectives into account. There is also need for a wider consideration of the consequences of using such technologies and the consequences of implementing significant healthcare intervention within the NHS without prior rigorous research. Specifically, the lack of financial analysis is an imperative next step for researchers to address in order to establish whether BWCs will provide a cost‐effective use of funding to improve mental health service delivery.

This review only included studies with public services actively utilising BWC technology. During the initial screening process, it was evident that many studies examined BWCs beyond the scope of this review. For example, several law enforcement studies which did not meet the inclusion criteria for this review relied on archival footage, which may provide a different insight into BWC outcomes in police settings. As the evidence base grows and follow‐up periods increase, it will be beneficial for researchers to compare pre/post data and consider possible therapeutic outcomes and unintended consequences in more depth. Additionally, future research into BWCs in mental health settings should prioritise co‐production and patient involvement, as this review highlighted a notable lack of consideration for patient and citizen perspectives in research.

## CONCLUSION

This review established that there is a poor evidence base for the use of BWCs in public sector services. BWCs in law enforcement is a well‐established practice with limited empirical support, and the increase in the use of this technology across other public services, such as healthcare, is still under‐researched. The use of poor‐quality law enforcement data to support the application of this technology in mental healthcare settings raises concerns around power and coercion in mental health nursing. This review highlights questions around the positive and negative impacts of BWCs in inpatient mental health settings have yet to be answered.

## RELEVANCE FOR CLINICAL PRACTICE

BWCs are actively being rolled out in mental health trusts across the UK without a substantial evidence base to support their use. With this growing implementation, it is surprising that there is such a dearth of research that considers patient voices. The current review highlights the need to explore the experiences and perspectives of patients, mental health staff, and senior management to better understand the motivations, concerns, barriers, impact, and unintended/adverse consequences of BWC use in mental health settings. This research will help the mental health sector gain a greater understanding of this complex issue to better inform policies and practice.

## Funding information

No external funding.

## Appendix A Summary of studies included in review

**Table:**

Author	Title	Country	Camera	BWC User	Study design	Quality
**Healthcare**						
Broach *et al*. (2018)	Usability and Reliability of Smart Glasses for Secondary Triage During Mass Casualty Incidents	USA	Google Glass	Paramedics	Cohort study	Medium
Ellis *et al*. (2019)	The Use of Body Worn Video Cameras on Mental Health Wards: Results and Implications from a Pilot Study	UK	Calla	Clinical Staff	Quasi‐experimental	Poor
Gupta *et al*. (2016)	Does Wearable Medical Technology with Video Recording Capability Add Value to On‐Call Surgical Evaluations?	USA	Google Glass	Physician	Cohort study	Medium
Hardy *et al*. (2017)	The Feasibility of Using Body Worn Cameras in an Inpatient Mental Health Setting	UK	Calla	Clinical staff	Quasi‐experimental	Poor
Ho *et al*. (2017)	Effect of Body‐Worn Cameras on EMS Documentation Accuracy: A Pilot Study	USA	TASER	Paramedics	Cohort study	Medium
Matthews *et al*. (2015)	Usability of a Wearable Camera System for Dementia Family Caregivers	USA	CMOS	Family Carer	Cross‐sectional	Medium
Noorian *et al*. (2019)	Use of Wearable Technology in Remote Evaluation of Acute Stroke Patients: Feasibility and Reliability of a Google Glass‐Based Device	USA	Google Glass	Physician	Cohort study	Good
Skolnik *et al*. (2016)	Tele toxicology: Patient Assessment Using Wearable Audio‐Visual Streaming Technology	USA	Google Glass	Physician	Cohort study	Medium
**Police**						
Ariel *et al*. (2015)	The effect of police body‐worn cameras on use of force and citizens' complaints against the police: A randomized controlled trial	USA	TASER	Police officer	RCT	Poor
Ariel (2016b)	Police body cameras in large police departments	USA	TASER	Police officer	Quasi‐experimental	Medium
Ariel *et al*. (2016)	Wearing body cameras increase assaults against officers and does not reduce police use of force: Results from a global multi‐site	Not specified	Unknown	Police officer	Cluster RCT	Poor
Ariel (2016a)	Increasing cooperation with the police using body worn cameras	USA	TASER	Police officer	Quasi‐experimental	Poor
Ariel *et al*. (2017)	“Contagious accountability” A global multisite randomized controlled trial on the effect of police body worn cameras on citizens' complaints against the police.	Not specified	Unknown	Police officer	RCT	Poor
Ariel *et al*. (2018)	Paradoxical effects of self‐awareness of being observed: Testing the effect of police body‐worn cameras on assaults and aggression against officers.	Not specified	Unknown	Police officer	Cluster RCT	Poor
Braga *et al*. (2018)	The effects of body‐worn cameras on police activity and police‐citizen encounters: A randomized controlled trial.	USA	TASER	Police officer	RCT	Medium
Braga *et al*. (2019)	Measuring the Direct and Spill over Effects of Body Worn Cameras on the Civility of Police‐Citizen Encounters and Police Work Activities	USA	Unknown	Police officer	RCT	Poor
Demir *et al*. (2018)	Body Worn Cameras, Procedural Justice, and Police Legitimacy: A Controlled Experimental Evaluation of Traffic Stops	Turkey	Unknown	Traffic officer	Quasi‐experimental	Medium
Demir (2019)	Citizens' perceptions of body‐worn cameras (BWCs): Findings from a quasi‐randomized controlled trial	Turkey	Unknown	Traffic officer	Quasi‐RCT	Medium
Demir *et al*. (2020)	The effect of body‐worn cameras on satisfaction and general perceptions of police: Findings from a quasi‐randomized controlled trial	Turkey	Unknown	Traffic officer	Quasi‐experimental	Medium
Ellis *et al*. (2015)	Evaluation of the introduction of personal issue body worn video cameras (Operation Hyperion) on the Isle of Wight: Final report to Hampshire Constabulary.	UK	Reveal	Police officer	Quasi‐experimental	Poor
Gaub *et al*. (2016)	Officer perceptions of body‐worn cameras before and after deployment: A study of three departments.	USA	Unknown	Police officer	Cross‐sectional	Poor
Gaub *et al*. (2020b)	One size does not fit all: The deployment of police body worn cameras to specialty units.	USA	Unknown	Police officer	Qualitative	Poor
Gaub *et al*. (2020a)	The distribution of police use of force across patrol and specialty units: A case study in BWC impact.	USA	Unknown	Police officer	Quasi‐experimental/RCT	Poor
George and Meadows (2016)	Policing on the Surveillance Frontier: Officer Perspectives of Body‐Worn Cameras	USA	Puma	Police officer	Cross‐sectional	Poor
Groff (2020)	The effects of body‐worn cameras on police‐citizen encounters and police activity: evaluation of a pilot implementation in Philadelphia	USA	Unknown	Police officer	Quasi‐experimental	Medium
Grossmith *et al*. (2015)	Police, Camera, Evidence: London’s cluster randomised controlled trial of Body Worn Video	UK	Unknown	Police officer	Cluster RCT	Poor
Headley *et al*. (2017)	A field experiment of the impact of body‐worn cameras (BWCs) on police officer behavior and perceptions	USA	TASER	Police officer	Quasi‐experimental	Poor
Hedberg *et al*. (2017)	Body‐worn cameras and citizen interactions with police officers: Estimating plausible effects given varying compliance levels	USA	VIEVU	Police officer	Quasi‐experimental	Poor
Henstock and Ariel (2017)	Testing the effects of police body‐worn cameras on use of force during arrests: A randomized controlled trial in a large British police force	UK	Reveal	Police officer	RCT	Good
Huff *et al*. (2020)	A randomized controlled trial of the impact of body‐worn camera activation on the outcomes of individual incidents.	USA	TASER	Police officer	RCT	Poor
Hyatt *et al*. (2017)	The effects of a mandatory body‐worn camera policy on officer perceptions of accountability, oversight, and departmental culture	USA	Unknown	Police officer	Cross‐sectional	Medium
Jennings *et al*. (2015)	Evaluating the impact of police officer body‐worn cameras (BWCs) on response‐to‐resistance and serious external complaints	USA	TASER	Police officer	RCT	Poor
Jennings *et al*. (2017)	A quasi‐experimental evaluation of the effects of police body‐worn cameras (BWCs) on response‐to‐resistance in a large metropolitan police department.	USA	Unknown	Police officer	Quasi‐experimental	Medium
Koen *et al*. (2019)	The effects of body‐worn cameras on police organisation and practice: A theory‐based analysis	USA	Unknown	Police officer	Qualitative	Good
Makin (2016)	When the watchers are watched: An interpretive phenomenological analysis of body‐worn cameras.	USA	TASER	Police officer	Qualitative	Good
Rankin (2013)	On‐officer body camera system: Program evaluation and recommendations.	USA	TASER	Police officer	Quasi‐experimental	Poor
Mitchell *et al*. (2018)	Measuring the effect of body‐worn cameras on complaints in Latin America: The case of traffic police in Uruguay	Uruguay	Reveal	Police officer	Quasi‐experimental	Good
Morrow *et al*. (2016	Assessing the impact of body‐worn cameras on arresting, prosecuting and convicting suspects of intimate partner violence.	USA	VIEVU	Police officer	Quasi‐experimental	Medium
ODS Consulting (2011)	Body worn video projects in Paisley and Aberdeen, self‐evaluation.	UK	Unknown	Police officer	Quasi‐experimental	Poor
Owens *et al*. (2014)	The Essex body worn video trial: The impact of body worn video on criminal justice outcomes of domestic abuse incidents	UK	Unknown	Police officer	RCT	Poor
Pelfrey and Keener (2016)	Police body worn cameras: A mixed method approach assessing perceptions of efficacy.	USA	Unknown	University police officer	Quasi‐experimental	Medium
Peterson *et al*. (2018)	The Milwaukee police department's body‐worn camera program: Evaluation findings and key takeaways	USA	TASER	Police officer	RCT	Poor
Pope *et al*. (2020)	Body Worn Video Camera (BWVC) Pilot Evaluation	UK	Unknown	Prison guards	Quasi‐experimental	Poor
Ready and Young (2015)	The impact of on‐officer video cameras on police–citizen contacts: Findings from a controlled experiment in Mesa	USA	TASER	Police officer	Quasi‐experimental	Poor
Roy (2014)	On‐officer video cameras: Examining the effects of police department policy and assignment on camera use and activation.	USA	TASER	Police officer	Quasi‐experimental	Poor
Sutherland *et al*. (2017)	Post‐experimental follow‐ups—Fade‐out versus persistence effects: Rialto police body‐worn camera experiment four years on	USA	TASER	Police officer	Quasi‐experimental	Poor
TPS Strategy Management (2016)	Body‐worn cameras: A report on the findings of the pilot project to test the value and feasibility of body‐worn cameras for police officers in Toronto	Canada	Reveal	Police officer	Quasi‐experimental	Poor
Wallace *et al*. (2018)	Body‐worn cameras as a potential source of de‐policing: Testing for camera‐induced passivity.	USA	Unknown	Police officer	RCT	Poor
White *et al*. (2018)	Exploring the potential for body‐worn cameras to reduce violence in police‐citizen encounters.	USA	TASER	Police officer	RCT	Poor
Yokum *et al*. (2017)	Evaluating the effects of police body‐worn cameras: A randomized controlled trial.	USA	Unknown	Police officer	RCT	Poor
Young and Ready (2016)	A Longitudinal Analysis of the Relationship between Administrative Policy, Technological Preferences, and Body‐Worn Camera Activation among Police Officers	USA	TASER	Police officer	Cross‐sectional	Medium
**Transportation**						
Ariel *et al*. (2019)	Reducing Assaults Against Staff Using Body‐Worn Cameras (BWCs) in Railway Stations	UK	TASER	Railway staff	Cluster RCT	Medium
